# Supplementary material for: Structures of two aptamers with differing ligand specificity reveal ruggedness in the functional landscape of RNA
Source: eLife. 2018 Jun 7;7:e36381. doi: 10.7554/eLife.36381 (PMC6031431; doi:10.7554/eLife.36381)
Supplement: Supplementary file 1. [file elife-36381-supp1.docx]

**TABLE S1**

| PDB: | 6CK5 | 6CK4 |
| --- | --- | --- |
| RNA | Wild type | G96A mutant |
| Data Collection: |  |  |
| Beamline | 24-IDE at APS | 24-IDE at APS |
| Space group | P 1 2_1_ 1 | P 1 |
| Unit cell: |  |  |
| a, b, c (Å) | 34.0, 89.6, 136.4 | 53.3, 62.1, 125.9 |
| α, β, γ (º) | 90.0, 96.9, 90.0 | 91.2, 89.6, 101.9 |
| Wavelength (Å) | 0.979180 | 0.979180 |
| Resolution (Å) | 40.00 – 2.50 (2.54-2.50) | 40.00 – 3.10 (3.15 – 3.10) |
| R_merge_ | 0.100 (1.068) | 0.100 (1.222) |
| R_pim_ | 0.061 (0.633) | 0.082 (1.026) |
| I/σI | 16.431 (1.27) | 8.36 (0.67) |
| CC_1/2_ in highest-resolution shell | 0.176 | 0.253 |
| CC* in highest-resolution shell | 0.547 | 0.635 |
| Completeness (%) | 97.0 (99.6) | 93.7 (94.7) |
| Redundancy | 3.6 (3.9) | 2.2 (2.1) |
| Total Reflections | 769056 | 49095 |
| Unique Reflections | 27671 | 28567 |
| Refinement: |  |  |
| Resolution (Å) | 37.35 – 2.50 | 36.54 – 3.10 |
| Number of reflections | 26309 | 26642 |
| R_work_/R_free_ | 0.215/0.252 | 0.248/0.304 |
| Number of atoms: |  |  |
| Total | 4545 | 9041 |
| RNA | 4418 | 8829 |
| Ligand | 44 | 144 |
| Cations | 31 | 39 |
| Water | 52 | 21 |
| B factors: |  |  |
| Overall | 83.9 | 144.6 |
| RNA | 84.1 | 145.3 |
| Ligand | 73.9 | 123.0 |
| Cations | 117.9 | 119.0 |
| Water | 62.3 | 95.6 |
| RMSD: |  |  |
| Bond lengths (Å) | 0.0086 | 0.018 |
| Bond angles (º) | 1.8271 | 1.852 |

*Table S1. Crystallography statistics from data collection and refinement for the wild type and G96A structures.*
